# Population attributable risks of neurobehavioral disorders due to low birth weight in US children

**DOI:** 10.12715/apr.2014.1.2

**Published:** 2014-11-28

**Authors:** Sandie U. Ha, Lindsay A. Thompson, Greg Kearney, Jeffrey Roth, Xiaohui Xu

**Affiliations:** 1 Department of Epidemiology, University of Florida, Gainesville, USA; 2 Department of Pediatrics, University of Florida, Gainesville, USA; 3 Department of Public Health, East Carolina University, Greenville, USA; 4 Department of Neonatology, University of Florida, Gainesville, USA

## Abstract

**Background:**

The prevalence of low birth weight (LBW) and neurobehavioral disorders (N) has increased over the last three decades. However, it is unclear how many excess cases of ND can be attributed to LBW among U.S. children. The objectives of this study were to a) determine the association between LBW and ND including attention deficit/hyperactivity disorder (ADHD), autism spectrum disorder (ASD), behavior and conduct disorder (BCD) and learning disability (LD); and b) determine the population attributable risk (PAR) of these disorders due to LBW.

**Methods:**

Study subjects were a nationally representative cross-sectional sample of 85,637 children ages 2 to 17 years old who participated in the 2011 National Survey of Children’s Health. Birth weight and ND were reported by parents.

**Results:**

LBW accounted for 6.0% (95% confidence interval [CI] 2.3–10.4) of all ASD cases, 2.4% (CI 0.1–5.1) of BCD, and 6.8% (CI 4.8–9.0) of LD among the study population. There was not a significant association between LBW and ADHD. The percentages of these ND due to LBW were found to be higher among those who were pre-term and among ethnic minority groups.

**Conclusions:**

Based on our results and given that over 8% of U.S. children are born with LBW, with higher rates among minorities and preterm births, prospective planning for neurobehavioral services is warranted. Efforts to reduce ND in children align with national efforts to reduce LBW.

## Introduction

Rates of low birth weight (LBW: birth weight less than 2,500 grams) have been increasing since the mid 1980s in the United States [1]. The rate of LBW in 2011 was 8.1 per 100 births [2] up from 6.8 per 100 in 1980s [3]. Numerous studies have shown that LBW is associated not only with mortality [4] but a myriad of adverse health outcomes, including decreased lung function [5], impaired renal function [6], cardiovascular abnormalities [7], and other complications [4]. As LBW-related neonatal and infant mortality have significantly declined over the past years due to improved perinatal care, other LBW-associated health outcomes became even more important public health issue due to the increased incidence of LBW and improved survival of LBW infants [1].

Like LBW, neurobehavioral (ND) and neurodevelopmental disorders, such as attention deficit disorder (ADD)/attention deficit and hyperactivity disorder (ADHD) and autism spectrum disorders (ASD) have also increased during the past several decades [8, 9]. Using nationally representative data, Boyle et al. found that during the last 12 years, the prevalence of autism and ADHD have increased by 290% and 33%, respectively [8]. In addition to requiring special services, such as special education and counseling, these neurologic disorders may compromise quality of life and incur high health care expenditures for patients and their families [10, 11]. LBW is a major risk factor for neurobehavioral disorders such as ADHD and autism [12, 13]. Nonetheless, to our knowledge. there have not been any studies conducted to estimate the population attributable risk associated with LBW and ND in U.S. children.

In this study, we used data from the 2011 National Survey of Children’s Health (NSCH) to examine this association. Specifically, the purpose of this study was to determine the strength of association between parent-reported LBW and ND in a nationally representative sample of children after adjustment for potential confounders. We then estimated the population-attributable risks and excess cases of these disorders due to LBW. The findings of this study provide information on the potential LBW-associated disease burden in the U.S. population and are useful for health and education program planning.

## Methods

### Data Source

Data for this study come from the 2011 NSCH, which was funded by the Maternal and Child Health Bureau of the Health Resources and Services Administration, and conducted by the Centers for Disease Control and Prevention, National Center for Health Statistics. The purpose of the NSCH is to provide national and state-specific prevalence rates of various health conditions and to assess the health and well-being of U.S. children in broad domains including medical care, housing, family interactions, parental health, school and after-school activities, safe neighborhoods and social connectedness. The 2011 NSCH was a random-digit-dial phone survey conducted between February 2011 and June 2012. One child was randomly selected per household. The respondent was identified by the interviewer as the parent or guardian most knowledgeable about the selected child’s health. The total sample size consisted of 95,677 children ages 0 through 17. The sample was stratified by state so that each state had approximately 1,800 children participating in the survey.

### Study population

For the purposes of this study, children 2 through 17 years old were selected because neurobehavioral questions were asked only amongst this age group, and are most functionally relevant to this age group. As a result of these exclusions, this analysis included 85,637 children ages 2 through 17 years old.

### Exposure and outcome measures

The main predictor variable of interest was parent-reported LBW. Birth weight was assessed through the question, “What was [sampling child’s] birth weight?” Children born at a birth weight less than 2,500 grams (5.5 pounds) were classified as LBW. Children with birth weight of 2,500 grams (5.5 pounds) or greater were classified as normal birth weight (NBW). The main outcomes of interest were parent-reported ND, which included ADHD, ASD, behavioral or conduct disorders (BCD), and learning disability (LD). The outcomes were assessed using the telephone survey responses. A child is defined as a case if his/her parent answered “yes” to the outcome-specific questions below:

- ADHD: “Has a doctor or other health care provider ever told you that [sampling child] had attention deficit disorder or attention deficit hyperactive disorder, that is, ADD or ADHD?”
- ASD: “Has a doctor or other health care provider ever told you that [sampling child] had autism, Asperger’s syndrome, pervasive developmental disorder, or other autism spectrum disorder?”
- BCD: “Has a doctor or other health care provider ever told you that [sampling child] had behavioral or conduct problems, such as oppositional defiant disorder or conduct disorder?”
- LD: “Has a doctor, health care provider, teacher, or school official ever told you [sampling child] had a learning disability?”

### Covariates

Covariates considered in the study included age group (2–5, 6–10, 10–13, and 14–17 years-old), gender (male, female), ethnicity (non-Hispanic white, non-Hispanic black, Hispanic or other), place of residence (metropolitan, non-metropolitan), family structure (two-parent biological, two-parent step family, single mother, or other), insurance status (yes or no), exposure to environmental smoking (yes or no), maternal education (less than high school, high school graduate, or greater than high school), primary language (English or other), poverty level (<100% federal poverty level (FPL), 100%–200% FPL, or more than 200% FPL), exposure to neighborhood violence (yes or no), presence of someone with mental illness in household (yes or no), presence of someone with alcohol/drug problems in the household (yes or no), prematurity status (yes or no).

### Statistical analysis

The 2011 NSCH employed a complex sampling design that involved unequal selection probability of children within households and households within states. Therefore, all analyses were performed with adjustment for sample weight to account for sampling design, potential non-coverage (for those who did not have a home phone line) and non-response bias. All analyses were performed using SAS 9.3 (SAS Institute, Cary, NC).

Chi-square tests were used to describe and compare the distribution of weighted prevalence of LBW measures among children with ascertained demographic characteristics (e.g. gender, age, race, maternal education, etc). In addition, the weighted prevalence of ND among children by demographic characteristics was also tested. Three logistic regression models were fitted, using PROC SURVEYLOGISTIC, to estimate the unadjusted and adjusted odds ratio (OR) and 95% confidence intervals (CI) for having ND, comparing children with various LBW measures to those of normal birth weight (NBW). The first model [model 1] fitted the unadjusted OR for neurobehavioral disorders. The second model [model 2] was a parsimonious model, which adjusted for potential confounders that are found to be associated with both LBW measures and neurobehavioral outcomes in Tables 1 and 2. The third model [model 3] adjusted for all epidemiologically relevant variables (race, gender, and age), as well as other variables that significantly contribute to the model with joint test p-value <0.10. All three models were adjusted for complex survey design sampling weight.

Population-attributable risk percentage (PAR%) was estimated for each of the parent-reported neurobehavioral disorders using Levine’s formula: PAR%=([(Pexp(OR−1)]/[ Pexp(OR−1)+1])× 100, where Pexp is the prevalence of exposure to LBW, and OR is the unadjusted OR for each disorder [14, 15]. We further calculated the number of excess ND cases attributed to various LBW measures by multiplying the PAR% by the number of weighted total cases for that condition. Since LBW rates are different by prematurity status and ethnic groups, we stratified this analysis by these variables to make the findings more informative from a public health perspective.

## Results

There were a total of 85,637 children, ages 2 through 17 years old in the 2011 NSCH survey. Among them, 4,530 had missing birth weight, leaving 81,107 available for analysis. Of these, 9.5% (SE: 0.23) were born with a LBW as reported by their parent, corresponding to 5.88 million children living in 2011 who were born with LBW in previous years (data not shown). Table 1 shows that children who were female, non-Hispanic black, had single mothers, had less educated mothers, were poorer, lacked insurance, were exposed to in-home smoking, or born prematurely were more likely to have LBW compared to those with normal BW.

The weighted prevalence of parent-reported ND among children ages 2 to 17 was approximately 9.9% for ADHD, 2.3% for ASD, 4.1% for BCD, and 10.6% for LD (for ages 3 through 17 years-old since this question was only asked amongst children in this age group). Among children 3 to 17 years-old, 10.5% had only 1 of the 4 ND, and 8.2% had 2 or more co-occurring disorders. When compared to NBW children, the prevalence for all ND was significantly higher among LBW children (Table 2). The prevalence of ND by contributory covariates are also presented in Table 2.

The ORs of the associations between parent-reported LBW and ND among U.S. children ages 2 through 17 years old are shown in Table 3. Unadjusted and multivariate logistic regression models indicated that LBW was associated with all ND, except ADHD (Table 3). Specifically, LBW children had a 1.77 times increased odds of having LD compared to those with NBW in the unadjusted model (OR: 1.77, 95%CI: 1.53–2.04). After adjusting for covariates, this association remained significant (adjusted OR (aOR) = 1.47; 95%CI: 1.21–1.78). For all other disorders, except ADHD, LBW appeared to significant associations in the unadjusted model; however, these associations became insignificant after adjustment for important covariates.

In order to obtain more refined birth weight exposure, we also investigated the risk for other LBW categories: moderately LBW (MLBW: between 1,500 and 2,499 grams) and very LBW (VLBW: less than 1,500 grams). Overall, the results remained consistent with VLBW having stronger associations with all disorders (data not shown). We further investigated the effects of LBW on co-occurrence of ND. Our results showed that children with LBW also have significantly higher odds of having two or more co-occurring disorders after adjustment for confounders (aOR: 1.28, 95% CI: 1.00, 1.64) (Table 3). Effect modification by gender and race were considered for all disorders. No statistically significant differences were found.

Table 4 shows the population-attributable risk percentages (PAR%) for parent-reported ND due to LBW among children between 2 and 17 years of age in the U.S. by race and preterm delivery (PTD) status. Among the four disorders, LBW was attributed to the highest PAR% for LD, followed by ASD, and BCD. No statistically significant association was found for ADHD. The percent of total cases attributed to LBW in the population was 6.8 (95% CI: 4.8–9.0) for LD, 6.0 (95% CI: 2.3–10.4) for ASD, and 2.4 (95% CI: 0.1–5.1) for BCD. These percentages correspond to approximately 448,467 excess cases of LD, 89,107 excess cases of ASD and 65,232 excess cases of BCD among children ages 2 through 17 years-old in the U.S in 2011, due to LBW.

We also stratified results by ethnicity and prematurity status (born 3 weeks prior to the due date as reported by parents) due to difference in prevalence of parent-reported LBW. Among non-Hispanic whites, the percent of total cases attributed to LBW in the population was 5.3 (95% CI: 3.8–7.1) for LD, 4.6(95% CI:1.8–8.2) for ASD, and 1.9 (95% CI:0.1–4.0) for BCD. These percentages correspond to 180,967 excess cases of LD, 41,500 cases of ASD and 25,348 excess cases of BCD in 2011. For these same disorders, the PAR% was higher among non-Hispanic black, Hispanic and other ethnicity groups compared to non-Hispanic whites. However, the number of excess cases was lower for these minorities groups because the population is smaller and thus the total number of cases was lower (Table 4).

Among pre-term births, the percent of total cases associated with LBW was significantly higher compared to full-term births (Table 4). For this population, PAR% for parent-reported ND due to LBW were 28.1 for LD, 25.3 for ASD and 11.7 for BCD.

## Discussion

This study found evidence that in 2011, U.S. children ages 2 through 17 years-old who were born having LBW were significantly more likely to have ASD, BCD and LD, but not ADHD, as reported by their parents. In effect, LBW was likely responsible for a considerable number of excess cases of ND reported during the study period. There was also a clear racial disparity in terms of the extent of effects that LBW has on ND. In particular, the proportions of each disorder attributed to LBW among non-Hispanic black, Hispanic, and other race/ethnicity groups were considerably higher than among non-Hispanic whites. Our findings further illustrate that the proportions of each disorder attributed to LBW among preterm births were significantly higher compared to full-term births.

The associations between parent-reported LBW and ND found in this study were consistent with previous studies that investigated effects of various LBW indicators on various neurobehavioral health outcomes [12, 13, 16, 17]. For example, a recent meta-analysis showed that children with VLBW have increased risk of having executive problems and lower academic achievement [18]. Another study investigated the association between moderate birth weight (2,500–2,999 grams) compared with birth weights of 3,500–3,999 grams and found that the moderate birth weight group had a 20% increased risk of LD [19]. This association is weaker than our findings perhaps due to the fact that the authors compared the lower range of NBW to the higher range of NBW in our definition. In another study, Stein et al. compared LBW to NBW children and found that all LBW infants, even those with only moderate LBW, had increased risks of learning and behavioral problems[20].

On the other hand, the lack of significant association between LBW and ADHD in our study is inconsistent with the literature [12, 19]. For example, in a recently study using a within-twin pair design, Pettersson et al. found that reduced birth weight is independently associated with ADHD symptoms measured by DSM-IV criteria [21]. Another study also found that birth weight is indirectly associated with ADHD severity through disruption of neuropsychological functions [22]. The reason for the discrepancy is unclear but is beyond the scope of our study. However, additional studies are warranted.

We stratified our analysis of PAR% by race and prematurity status because historically the prevalence of LBW has been consistently different for these groups. These analyses help address the question whether the higher prevalence of ND among ethnic minority groups and premature births can be explained by the higher proportion of LBW. It appears that LBW has the strongest effects among ethnic minorities because although the number of excess cases is lower among them (due to smaller population), their attributable risks are higher compared to non-Hispanic whites for every disorder. Further analyses also showed very high numbers of excess cases for each ND, in particular LD and ASD. These findings suggest that if LBW can be reduced, fewer ND would be diagnosed in US children ages 2 through 17 years-old. More importantly, the higher PAR among minorities suggests that if LBW could be reduced in these groups in which it is more prevalent, the gap in pediatric neurobehavioral outcomes between non-Hispanic white and other ethnic groups would decrease.

The higher PAR% of parent-reported ND due to LBW among preterm births also suggest that a higher proportion of cases among this group is possibly due to LBW. This finding also implies that if preterm births can be reduced, there would be fewer excess cases of ND. While there are myriad factors that influence LBW that cannot be controlled, one potential means of decreasing preterm births is to reduce the rates of elective induction of labor. Given the prevalence of elective labor induction prior to due date in the United States [23, 24], it is now important to reconsider this procedure in light of its impact on excess neurobehavioral disorders as well as other adverse birth outcomes.

Maternal malnutrition and placental insufficiencies are two known factors that influence LBW [25]. While maternal malnutrition is not likely to be a widespread problem in developed countries such as the U.S., food insufficiency remains a problem and is addressed by programs including the Special Supplemental Nutrition Program for Women, Infants, and Children (WIC) and the Supplemental Nutrition Assistance Program (“food stamps”). Since more than 50% of pregnancies are unplanned, physicians and public health officials need to educate reproductive age women about the importance of proper nutrition prior to and during pregnancy [26, 27]. The second mechanism that affects LBW, placental insufficiency, is currently not well understood. Among the many contributors to LBW [28], there is evidence that exposure to environmental pollution can affect the maternal-fetal exchange mechanisms [29, 30] and increase the risk of adverse birth outcomes including LBW [30–32]. Therefore, reduction in exposure to pollution may improve the outcomes.

This study has several strengths. First, the sample is generally applicable to U.S. children because it utilizes data from a nationally representative sample of children from ages 2–17. Second, the study is the first to assess PAR% and excess cases of ND due to LBW. However, the study also has several limitations. First, both exposure and outcome are based on parental reporting, and thus the information may not represent actual diagnoses. Consequently, misclassification bias may have occurred. For example, our analysis showed a higher LBW prevalence compared to the national U.S. average of 8.1% during 2011 [33]. This higher prevalence may reflect the inaccurate recall of birth weight by a parent and is an inherent limitation of parental reported health information without validation. It is also possible that parents with LBW children are more likely to remember and report birth weight. One validation study, using a different data source, evaluated consistency and completeness of maternally reported information related to birth outcomes (LBW) and found that this information was consistent with birth certificate records [34]. Furthermore, approximately 6% of children in the study sample had missing birth weight values, another source of potential bias. We assessed the proportion of missing birth weight values and found that ethnic minorities and older children are more likely to not have birth weight listed.

Secondly, since this study relied on available data, we were unable to adjust for all possible confounders. For example, maternal age at delivery, gestational age, and pregnancy complications could be important confounders; however, this information was not available for analysis. The lack of information about these covariates prevented us from examining other potential reasons for LBW, such as congenital anomalies or genetic disorders, that also lead to neurobehavioral deficits and disorders.

Although this is a cross-sectional study, temporality can be assumed because it is not logical to claim NBD precede LBW. We also acknowledge the limitation of using OR as an estimate of risk. When used in Levine’s formula, OR may have overestimated the attributable risk. However, given the low prevalence of ND, overestimation is not likely to be a serious concern.

## Conclusions

The study showed that parent-reported LBW is a major contributor to parent-reported pediatric ND including LD, ASD and BCD. Decreasing the occurrence of LBW births could potentially reduce the number of ND in children between the ages of 2 and 17 in the U.S. However, since there is currently no effective population-level LBW prevention strategy, prospectively planning for neurobehavioral services is merited. Early referral would be particularly helpful for racial minority groups because the prevalence of LBW is higher in these groups compared to the white population. The findings of our study also suggest that efforts to prevent neurodevelopment disorders in children need to start prior to conception and be maintained throughout pregnancy to assure that the infant is born at full-term.

## Figures and Tables

**Table 1:** Birth weight status and characteristics among children aged 2–17 in the United States, 2011 (n=85,637)

Characteristics	Low Birth Weight (LBW: <2500g) N (weighted %) or Mean (SD)		
	LBW 7402(9.5)	NBW 73705(90.5)	p-value^a^
Child age (year)			0.0525
2–5	1861(10.5)	17475(89.5)	
6–10	2379(9.2)	22013(90.8)	
10–13	1364(9.7)	13778(90.3)	
14–17	1798(8.8)	20439(91.2)	
Gender			< .0001
Male	3521(8.5)	38305(91.5)	
Female	3871(10.6)	35321(89.4)	
Race			< .0001
Non-Hispanic white	4063(7.3)	49152(92.7)	
Non-Hispanic black	1146(16.2)	6299(83.8)	
Hispanic	705(10.2)	6054(89.8)	
Others	1295(10.1)	10594(89.9)	
Place of residence			0.5323
Metropolitan	3990(10.0)	38902(90.0)	
Non-metropolitan	3329(9.4)	34137(90.6)	
Family Structure			< .0001
Two parents-biological	4569(8.6)	51463(91.4)	
Two parents-step	585(9.1)	5291(90.9)	
Single mother	1485(12.1)	11643(87.9)	
Others	669(11.6)	4492(88.4)	
Maternal education			0.0458
Less than HS	1235(10.5)	9688(89.5)	
HS graduate	2400(9.5)	24156(90.5)	
More than HS	3285 (8.9)	35959(91.1)	
Primary language			0.0640
English	6720(9.3)	68534(90.7)	
Others	674(10.7)	5136(89.3)	
Poverty level			< .0001
Below 100%	1372(11.9)	9232(88.1)	
100–200%	1366(10.2)	11743(89.8)	
Above 200%	3991(8.1)	46124(91.9)	
Insurance status			< .0001
No	359(13.0)	3068(87.0)	
Yes	7034(9.4)	70538(90.6)	
Exposure to ETS			0.0216
No	5313(9.2)	56233(90.8)	
Yes	2015(10.6)	16812(89.4)	
Neighbourhood violence			0.3552
No	6600(9.5)	66641(90.5)	
Yes	659(9.3)	5782(90.7)	
Household with mental illness			0.1099
No	6486(9.5)	65808(90.5)	
Yes	773(9.1)	6685(90.9)	
Household with alcohol/drug problems			0.3153
No	6352(9.6)	64833(90.4)	
Yes	916(9.0)	7740(91.0)	
Prematurity			< .0001
No	2542(4.1)	69039(95.9)	
Yes	4799(50.9)	4503(49.1)	

Abbreviations: ETS, Environmental tobacco smoke;

a Chi-square p-value for categorical variables and t-test p-value for continuous variables

**Table 2:** Prevalence of parent-reported neurobehavioral disorders by covariates among children ages 2 to 17 (n=85,637)

Characteristics	ADHD n (weighted %) 8528(9.9)		Autistic Spectrum Disorders (ASD) n (weighted %) 2041(2.3)		Behavior or conduct disorders (BCD) n (weighted %) 3236(4.1)		Learning Disability^a^ (LD) n (weighted %) 8324(10.6)		1 disorder^a^ n (weighted %) 7705(10.5)		2 or more co-occurring disorders^a^ n (weighted %) 5889(8.2)	
LBW		p=0.0949		p=0.0004		p=0.0360		p<0.0001		p<0.0001		p<0.0001
Yes	950(10.8)		263(3.6)		369(4.9)		1153(16.2)		911(13.3)		729(11.2)	
No	6438 (89.2)		7125 (96.4)		7028(95.1)		5930(83.8)		5415(86.7)		5415(88.8)	
Prematurity		p<.0001		p<.0001		p<.0001		p<.0001		p<.0001		p<.0001
No	7013(9.3)		1630(2.0)		2589(3.9)		6638(9.6)		6384(9.9)		4730(7.4)	
Yes	1331(13.2)		372(4.2)		540(5.5)		1519(17.4)		1199(14.7)		1015(13.3)	
Child age (year)		p<.0001		p=0.0016		p <.0001		p <.0001		p <.0001		p<.0001
2–5	344(2.3)		296(1.6)		251(1.7)		639(4.7)		608(4.0)		343(3.0)	
6–10	2513(9.7)		707(2.5)		1076(4.5)		2439(10.2)		2355(10.2)		1770(7.8)	
10–13	2199(13.7)		468(2.9)		743(5.0)		1981(13.0)		1802(12.7)		1480(10.8)	
14–17	3472(14.6)		570(2.3)		1166(5.3)		3265(13.7)		2940(14.5)		2296(11.0)	
Gender		p<.0001		p<.0001		p<.0001		p<.0001		p<.0001		p<.0001
Male	5940(13.5)		1618(3.6)		2204(5.5)		5419(13.5)		4878(13.2)		4150(11.7)	
Female	2585(6.1)		422(0.9)		1030(2.6)		2895(7.6)		2819(7.8)		1735(4.7)	
Race		p<.0001		P=0.0242		p= 0.0232		p= 0.0775		p=0.0022		p<.0001
Non-Hispanic white	5886(11.7)		1401(2.7)		1898(4.0)		5236(10.7)		5088(11.4)		3848(9.1)	
Non-Hispanic black	833(10.9)		172(2.1)		456(5.5)		888(12.2)		737(10.9)		648(9.7)	
Hispanic	542(7.0)		137(2.1)		258(4.0)		719(10.4)		606(8.9)		421(7.0)	
Others	1106(6.5)		294(1.7)		546(3.7)		1290(9.7)		1115(9.2)		847(5.9)	
Place of residence		p=0.0112		p=0.0060		p= 0.3171		p=0.4470		p=0.0768		p=0.2283
Metropolitan	4555(9.7)		1115(2.4)		1668(4.1)		4263(10.5)		4012(10.3)		3104(8.1)	
Non-metropolitan	3899(10.8)		911(1.9)		1544(4.4)		3985(10.9		3613(11.1)		2740(8.7)	
Family Structure		p<.0001		p=0.0636		P <,0001		p <.0001		p<.0001		p<.0001
Two parents-biological	4340(7.3)		1208(2.1)		1205(2.2)		4624(8.7)		4393(8.4)		2930(5.9)	
Two parents-Step	1089(17.2)		169(2.5)		438(7.8)		845(12.3)		865(16.3)		689(12.3)	
Single mother	1934(13.1)		463(2.9)		950(7.2)		1832(14.3)		1557(13.1)		1444(12.3)	
Others	1054(14.7)		175(2.1)		595(8.1)		921(14.7)		815(15.1)		744(12.4)	
Maternal education		p=0.0015		p=0.4510		p= <.0001		p= <.0001		p=0.1420		p=0.0060
Less than HS	1260(8.7)		287(2.2)		666(5.0)		1495(12.7)		1224(11.1)		984(8.2)	
HS graduate	3076(11.0)		665(2.4)		1133(4.4)		2853(11.0)		2679(10.9)		2065(9.1)	
More than HS	3646(9.6)		984(2.4)		1160(3.3)		3385(9.1)		3311(9.9)		2437(7.4)	
Primary language		p<.0001		p=0.0013		p= 0.0008		p= 0.0068		p<.0001		p<.0001
English	8340(11.2)		1969(2.5)		3081(4.4)		7793(10.9)		7248(11.1)		5707(9.1)	
Others	185(2.6)		72(1.0)		153(2.4)		525(8.6)		449(7.5)		181(3.0)	
Poverty level		p<.0001		p=0.6451		p <.0001		p <.0001		p<.0001		p<.0001
Below 100%	1496(12.1)		342(2.2)		888(7.2)		1714(15.6)		1286(13.5)		1217(11.6)	
100–200%	1635(10.7)		396(2.4)		759(4.9)		1672(11.4)		1456(11.1)		1212(9.4)	
Above 200%	4714(9.2)		1151(2.5)		1338(2.7)		4171(8.7)		4268(9.4)		2990(6.9)	
Insurance status		P=0.0001		p=0.0011		p= 0.0209		p= 0.1045		p=0.0565		p=0.0002
No	269(6.3)		50(1.0)		138(2.9)		323(8.8)		317(8.5)		182(4.8)	
Yes	8244(10.1)		1989(2.4)		3093(4.2)		7989(10.7)		7381(10.7)		5697(8.4)	
Exposure to ETS		p<.0001		p=0.1585		p <.0001		p <.0001		p<.0001		p<.0001
No	5606(8.3)		1480(2.2)		1873(3.1)		5617(9.4)		5294(9.2)		3815(6.8)	
Yes	2844(14.8)		546(2.6)		1329(7.2)		2625(14.3)		2347(14.7)		2015(12.8)	
Neighborhood violence		p<.0001		p=0.0096		p <.0001		p <.0001		p<.0001		p<.0001
No	6699(8.7)		1702(2.2)		2133(3.0)		6602(9.5)		6392(9.7)		4453(7.0)	
Yes	1622(20.8)		299(3.0)		996(14.1)		1521(20.2)		1148(18.4)		1282(20.1)	
Household with mental illness		p<.0001		p<.0001		p <.0001		P <.0001		p<.0001		p<.0001
No	6618(8.7)		1617(2.1)		2214(3.3)		6579(9.7)		6297(9.8)		4431(7.0)	
Yes	1692(21.1)		380(4.4)		925(12.4)		1538(19.5)		1226(18.1)		1305(20.5)	
Household with alcohol/drug problems		p<.0001		p= 0.0410		p <.0001		p <.0001		p<.0001		p<.0001
No	6347(8.7)		1668(2.2)		2078(3.3)		6456(9.7)		6092(9.6)		4314(7.1)	
Yes	2026(19.2)		345(2.9)		1089(10.5)		1718(17.1)		1471(17.7)		1469(17.0)	

Abbreviations: ADHD, attention deficit hyperactivity disorder; ETS, Environmental tobacco smoke

a Only analyzed for children ages 3 to 17

**Table 3:** Odds ratios for the association between low birth weight and neurobehavioral disorder in the United States, 2007

LBW categories	Model		Neurobehavioral disorders					
			ADHD	Autism Spectrum Disorder (ASD)	Behavior or conduct disorders (BCD)	Learning disability*(LD)	1 disorder*	2 or more co-occurring disorders*
LBW	Model 1^a^	No	Reference	Reference	Reference	Reference	Reference	Reference
		Yes	1.133 (0.979, 1.312)	**1.664 (1.249, 2.216)**	**1.260 (1.015,1.564)**	**1.769 (1.534, 2.040)**	**1.377 (1.176,1.611)**	**1.500 (1.267, 1.776)**
	Model 2^b^	No	Reference	Reference	Reference	Reference	Reference	Reference
		Yes	1.021 (0.836, 1.245)	1.356 (0.926, 1.985)	0.998 (0.756, 1.317)	**1.416 (1.169, 1.716)**	1.217 (0.994,1.490)	1.193 (0.940, 1.514)
	Model 3^c^	No	Reference	Reference	Reference	Reference	Reference	Reference
		Yes	1.069 (0.873, 1.310)	1.416 (0.962, 2.083)	1.070 (0.805, 1.423)	**1.467 (1.208, 1.783)**	**1.237 (1.004,1.523)**	**1.281 (1.004, 1.636)**

Bold font indicates statistical significance at alpha=0.05

Abbreviations: ADHD, attention deficit hyperactivity disorder; LBW, low birth weight

* only evaluated for children 3–17 years old

a Adjusted for complex survey design

b Adjusted for complex survey design and potential confounders that are significantly associated with both low birth weight and neurobehavioral outcomes according to Tables 1 and 2.

c Adjusted for complex survey design; epidemiologically relevant variables race, gender, age; and all other significant predictors (at p=0.10) in the model.

d Learning disability was restricted to only children from 3 to 17. Other disorders were for children from 2 to 17.

**Table 4:** Population-attributable risk (PAR) percentage for neurobehavioral disorders according to LBW by race and preterm delivery status, 2011

Race	Disorders^a^	Odds ratio (OR)	Percent exposed to LBW, P_exp_	PAR, %(95%CI)^b^	Total weighted cases	Excess cases^c^	Lower 95% CI	Upper 95% CI
Overall	ADHD	1.133(0.979, 1.312)	9.53	1.3(−0.2–2.9)	6,469,137	80,969	−12,973	186,797
	ASD	**1.664(1.249, 2.216)**	**9.53**	**6.0(2.3–10.4)**	**1,497,255**	**89,107**	**34,706**	**155,490**
	BCD	**1.260(1.015,1.564)**	**9.53**	**2.4(0.1–5.1)**	**2,697,896**	**65,232**	**3,851**	**137,613**
	LD	**1.769(1.534, 2.040)**	**9.53**	**6.8(4.8–9.0)**	**6,567,897**	**448,467**	**318,056**	**592,258**
By ethnicity								
Non-Hispanic white	ADHD	1.133(0.979, 1.312)	7.3	1.0(−0.2–2.2)	3,962,219	38,099	−6,083	88,234
	ASD	**1.664(1.249, 2.216)**	**7.3**	**4.6(1.8–8.2)**	**897,666**	**41,500**	**16,026**	**73,187**
	BCD	**1.260(1.015,1.564)**	**7.3**	**1.9(0.1–4.0)**	**1,360,856**	**25,348**	**1,489**	**53,814**
	LD	**1.769(1.534, 2.040)**	**7.3**	**5.3(3.8–7.1)**	**3,404,634**	**180,967**	**127,740**	**240,241**
Non-Hispanic black	ADHD	1.133(0.979, 1.312)	16.2	2.1(−0.3–4.8)	955,104	20,145	−3,260	45,952
	ASD	**1.664(1.249, 2.216)**	**16.2**	**9.7(3.9–16.5)**	**183,499**	**17,822**	**7,115**	**30,199**
	BCD	**1.260(1.015,1.564)**	**16.2**	**4.0(0.2–8.4)**	**478,492**	**19,340**	**1,160**	**40,059**
	LD	**1.769(1.534, 2.040)**	**16.2**	**11.1(8.0–14.4)**	**1,022,991**	**113,324**	**81,451**	**147,502**
Hispanic	ADHD	1.133(0.979, 1.312)	10.2	1.3(−0.2–3.1)	638,993	8,553	−1,372	19,708
	ASD	**1.664(1.249, 2.216)**	**10.2**	**6.3(2.5–11.0)**	**195,665**	**12,411**	**4,846**	**21,591**
	BCD	**1.260(1.015,1.564)**	**10.2**	**2.6(0.2–5.4)**	**362,012**	**9,353**	**553**	**19,693**
	LD	**1.769(1.534, 2.040)**	**10.2**	**7.3(5.2–9.6)**	**896,799**	**65,227**	**46,324**	**86,009**
Others	ADHD	1.133(0.979, 1.312)	10.1	1.3(−0.2–3.1)	790,050	10,472	−1,679	24,135
	ASD	**1.664(1.249, 2.216)**	**10.1**	**6.3(2.5–10.9)**	**199,048**	**12,510**	**4,883**	**21,772**
	BCD	**1.260(1.015,1.564)**	**10.1**	**2.6(0.2–5.4)**	**447,491**	**11,450**	**677**	**24,117**
	LD	**1.769(1.534, 2.040)**	**10.1**	**7.2(5.1–9.5)**	**1,103,675**	**79,543**	**56,479**	**104,910**
By PTD status								
Full term	ADHD	1.133(0.979, 1.312)	4.1	0.5(−0.1–1.3)	5,373,655	29,144	−4,631	67,872
	ASD	**1.664(1.249, 2.216)**	**4.1**	**2.7(1.0–4.7)**	**1,163,601**	**30,838**	**11,759**	**55,258**
	BCD	**1.260(1.015,1.564)**	**4.1**	**1.1(0.1–2.3)**	**2,225,510**	**23,474**	**1,368**	**50,300**
	LD	**1.769(1.534, 2.040)**	**4.1**	**3.1(2.1–4.1)**	**5,247,728**	**160,398**	**112,432**	**214,612**
Preterm	ADHD	1.133(0.979, 1.312)	50.9	6.3(−1.1–13.7)	978,965	62,071	−10,577	134,162
	ASD	**1.664(1.249, 2.216)**	**50.9**	**25.3(11.2**–**38.2)**	**314,027**	**79,324**	**35,323**	**120,057**
	BCD	**1.260(1.015,1.564)**	**50.9**	**11.7(0.8–22.3)**	**410,605**	**47,989**	**3,111**	**91,583**
	LD	**1.769(1.534, 2.040)**	**50.9**	**28.1(21.4–34.6)**	**1,225,024**	**344,612**	**261,808**	**424,020**

Bold face indicates statistical significance at alpha=0.05

Abbreviations: ADHD, attention deficit/hyperactivity disorder; ASD, autistic spectrum disorder; BCD, behavioral conduct disorder; LD, learning disability; PTD, pre-term delivery

a ADHD/ADD, Behavioral or Conduct disorders and Autistic spectrum disorders are only assessed among children ages 2 to 17. Learning disability is assessed among children 3 to 17.

b PAR%=([(P_exp_(OR−1)]/[P_exp_(OR−1)+1])× 100

c Excess cases = PAR% × total weighted cases
